# Association of Fecal Markers of Environmental Enteric Dysfunction with Zinc and Iron Status among Children at First Two Years of Life in Bangladesh

**DOI:** 10.4269/ajtmh.17-0985

**Published:** 2018-06-11

**Authors:** Shah Mohammad Fahim, Subhasish Das, Kazi Istiaque Sanin, Md. Amran Gazi, Mustafa Mahfuz, M. Munirul Islam, Tahmeed Ahmed

**Affiliations:** Nutrition and Clinical Services Division, International Center for Diarrheal Disease Research, Bangladesh (icddr,b), Dhaka, Bangladesh

## Abstract

Environmental enteric dysfunction (EED) causes gut inflammation and increased intestinal permeability leading to deficiencies in micronutrients such as zinc and iron. Fecal markers such as myeloperoxidase (MPO), neopterin (NEO), and alpha-1-anti-trypsin (AAT) can predict EED. The aim of this study was to examine the association between fecal markers of EED with zinc and iron status among children at first 2 years of life. Malnutrition and Enteric Disease Study Bangladeshi birth cohort data were used to conduct this analysis. Multivariable analyses using generalized estimating equations were performed to test the association between individual fecal markers with zinc or iron status of the children. A total of 265 children were enrolled in the study (male:female = 1:1). Of the 627 stool samples collected (*N* = 222 children), 535, 511, and 577 were accompanied by zinc, ferritin, and soluble transferrin receptor values, respectively. Median (interquartile range [IQR]) values of AAT, MPO, and NEO were 0.33 (0.18–0.62) mg/g, 3,895.42 (1,563.76–8,432.82) ng/mL, and 890.81 (331.57–2,089.04) nmol/L, respectively. Overall, 60%, 71%, and 97% of samples were above the values considered normal in nontropical settings for AAT, MPO, and NEO, respectively. High AAT levels were significantly associated with low ferritin values after adjusting for age and gender (coefficient = −5.85; 95% confidence interval = −11.23 to −0.47; *P* value = 0.03). No such association was found between AAT and plasma zinc status. Myeloperoxidase and NEO were not associated with plasma zinc or iron status. The study results imply the importance of enteric protein loss in contributing to reduced ferritin levels at first 2 years of life.

## INTRODUCTION

Stunting or linear growth faltering (length-for-age *z*-score [LAZ] < −2) is a global public health concern and highly prevalent in low- and middle-income countries. More than a third of children aged less than 5 years in South Asia and Sub-Saharan Africa are stunted or short for their age.^1^ Stunting implicates as great as 5-fold increased risk of mortality among under-5 children. It is even more perilous for the younger children aged less than 2 years.^2^ The period encompassing first 2 years of life is eminent for growth and development. If stunting continues beyond this period, much of its effect is irreversible, resulting in loss of human potential and reduced productivity in later life.^3^ According to Bangladesh Demographic and Health Survey 2014, 36% of < 5 children in Bangladesh are stunted.^4^ There are multiple etiologies and risk factors that contribute to stunting in children. Environmental enteric dysfunction (EED) and zinc deficiency are considered as the key determinants of linear growth failure in early years of life.^5,6^

Environmental enteric dysfunction, previously known as tropical enteropathy or environmental enteropathy, is a sub-clinical intestinal disorder that is significantly implicated with linear growth faltering.^7^ Environmental enteric dysfunction is thought to occur from poor hygiene and unsanitary environmental conditions leading to repeated enteric infections, which in turn results in intestinal inflammation, reduced absorptive capacity, disrupted barrier function, and ultimately leaky gut syndrome.^5,8,9^ Environmental enteric dysfunction demonstrates histological changes in the gut characterized by blunting of villi, increased depth of crypts, lymphocytic infiltration, and reduction in mucosal surface area.^5,10^ The gold standard for diagnosing EED is histopathology of biopsied specimen.^11^ But endoscopy for obtaining biopsy specimen is an invasive procedure and not feasible for children. Therefore, some candidate biomarkers are of interest that can predict gut inflammation and increased intestinal permeability caused by EED.^5^

Myeloperoxidase (MPO), neopterin (NEO), and alpha-1-anti-trypsin (AAT) are potential fecal markers for evaluation of EED among children.^12^ Myeloperoxidase and NEO indicate gut inflammation, whereas AAT is an useful marker of intestinal permeability.^13^ Myeloperoxidase is a specific marker of polymorphonuclear leukocyte activity. Elevated MPO level in stool reflects the inflammatory activity of Crohn’s disease or ulcerative colitis.^14^ Neopterin is the product of breakdown of cyclic guanosine monophosphate and indicates the inflammatory immune response of intestinal epithelium.^15^ Alpha-1-anti-trypsin is an acute-phase protein that is resistant to intestinal proteolysis and excreted intact in stool. Alpha-1-anti-trypsin is a classic marker of protein-losing enteropathy.^16^ Presence of AAT in stool indicates the increased intestinal permeability and protein loss.^12,13^ Based on the aforementioned biomarker levels, a composite EED score was developed for accurate prediction of EED and its subsequent consequences.^12,13^

Environmental enteric dysfunction also leads to altered absorption and metabolism of micronutrients such as iron and zinc.^17^ Iron and zinc are essential trace elements and plays important role in various enzyme systems related to growth and development in human body.^18^ Iron deficiency is the most common and widespread nutritional disorder in the world.^19^ In Bangladesh, 10.7% under-5 children are iron deficient and the prevalence is 27.2% in slum areas.^20^ Iron deficiency results in impaired physical development and anemia. More than 50% of anemia is due to iron deficiency. Anemia due to iron deficits affects cognitive and motor development of children and also causes low productivity in later life.^21^

On the other hand, globally, one-third of the children are at risk of zinc deficiency, and it accounts for 4.4% of child death worldwide.^22^ Nationwide estimates revealed that 44.6% under-5 children are zinc deficient in Bangladesh.^20,23^ The estimates were based on plasma zinc concentration which is a proxy marker for assessing zinc status. Although this measure is not reliable at the individual level because of low sensitivity, World Health Organization (WHO), United Nations Children’s Fund, and the International Zinc Nutrition Consultative Group jointly recommended the use of plasma zinc concentration for assessment of population zinc status.^24,25^ However, studies have reported that zinc deficiency is associated with increased risk of illness, impaired cognitive function, and stunting.^26–28^ In addition, zinc deficits result in decreased linear growth velocity in infants and growth retardation among children.^29^ Children suffering from EED have increased fecal loss of zinc, leading to decreased intestinal ion transport and altered mucosal immune function.^25,30^ Observational studies conducted in rural Malawi illustrated association between fecal loss of zinc with EED.^31^ The investigators carried out a study on 25 children at risk of EED due to poor hygiene or previous history of malnutrition and found that urinary biomarker of EED were positively associated with endogenous fecal zinc excretion.^32^

But till date, very little is known about the contribution of EED on iron and zinc deficits and its consequences such as mortality, morbidities, and linear growth faltering on children, especially during first 2 years of life. The aim of this study was to examine the association between fecal markers of EED (MPO, NEO, and AAT) with plasma iron and zinc status in children aged 2 years living in a slum in Bangladesh.

## MATERIALS AND METHODS

### Study design.

Malnutrition and Enteric Disease Study (MAL-ED) birth cohort data from Bangladesh site was used to conduct this analysis. The detailed methodology of the study has been published previously.^33^ The study protocol was reviewed and approved by the Ethical Review Committee of the International Center for Diarrheal Disease Research, Bangladesh (icddr,b). Informed written consent was obtained from the parents or legal guardians of the participants enrolled in the study.

### Study site and population.

The study was conducted in Bauniabadh area of Mirpur, an urban settlement in Dhaka, Bangladesh, with low socioeconomic conditions and suboptimal sanitation. Overall, 265 healthy newborns living in Bauniabadh area were enrolled in the study within first 17 days of life between February 2010 and February 2012. Exclusion criteria for cohort recruitment were maternal age of < 16 years, not a singleton pregnancy, another child already enrolled in the MAL-ED study, severe disease requiring hospitalization before recruitment, and severe acute or chronic conditions diagnosed by a physician (e.g., neonatal disease, renal disease, chronic heart failure, liver disease, cystic fibrosis, and congenital conditions).

### Data collection.

Assessment of socioeconomic and household information was carried out at enrollment. Enrolled participants were visited by MAL-ED field staffs every other day. Field assistants interviewed the parents or caregiver using structured and pretested questionnaire. Stool and blood samples were collected at 7, 15, and 24 months of age. Stool samples were collected without fixative by trained health workers and frozen at −70°C pending processing. Plasma was obtained via centrifugation of the blood. In this study, plasma zinc was assessed as the measure of zinc status. Plasma zinc concentration is a proxy marker and recommended to use for assessment of population zinc status, especially for children in low-income countries.^24,34^ In this study, the participants received zinc treatment of diarrhea as per the guideline of WHO^9^; therefore, the blood sample was collected only after they have recovered from diarrhea. However, whether the child was receiving zinc supplement during collection of blood was not monitored and documented. Plasma ferritin levels and soluble transferrin receptor (sTfr) values were measured to evaluate the iron status of the children. Ferritin indicates the measure of iron stores in the body if there is no concurrent infection. Soluble transferrin receptor reflects the intensity of erythropoiesis and the demand for iron in the body.^35^ Iron deficiency was defined as ferritin level less than 12 μg/L and zinc deficiency was considered when plasma zinc concentrations were < 9.9 mmol/L.^20^ Dietary data were obtained at 15 and 24 months of age using 24-hour recall method. A structured form was developed to record the food items and recipes that were offered to the child for consumption in previous 24 hours. The detailed methodology of dietary data collection was published elsewhere.^36^

### Laboratory analyses.

All laboratory analyses were performed in the laboratories at icddr,b in Dhaka, Bangladesh. Alpha-1-anti-trypsin (Biovendor, Chandler, NC), NEO (GenWay Biotech, San Diego, CA), and MPO (Alpco, Salem, NH) were measured in the stool samples using commercially available enzyme-linked immunosorbent assay kits, following the manufacturer’s instructions. Plasma zinc was measured by atomic absorption spectometry method. Ferritin and sTfR levels were measured using chemiluminescence immunoassay and immunoturbidimetry method, respectively.

### Statistical analyses.

Statistical analyses were performed using SPSS version 20.0 (IBM Corporation, Armonk, NY). To summarize the data, proportion estimate was used for categorical variables and median estimate with interquartile range (IQR) was used for asymmetric quantitative variables. Kruskal–Wallis test was applied for comparing nonparametric variables over months. Fecal marker concentrations were categorized based on the distribution of all measurements: low (in first quartile), medium (within the IQR), or high (in fourth quartile). At each time point, the composite EED score ranging from 0 to 10 was calculated from the three fecal markers, as described in the previous literature by MAL-ED co-investigators.^12,13^ Categories were assigned values as 0 (low), 1 (medium), or 2 (high). The formula for the composite EED score is as follows:

EED score = 2 × AAT category + 2 × MPO category + 1 × NEO category.

In this study, individual fecal marker or the composite EED score was the predictor and plasma zinc or ferritin or sTfR value was considered as outcome. The relationship between individual fecal marker and EED score with the zinc and iron status were examined separately using generalized estimating equation (GEE) model where the child was the cluster for the GEE model. Covariates for multivariable models were selected if their association with the outcome had significance < 0.2. The ferritin and zinc values were adjusted with the indicators of infection C-reactive protein and alpha-1-acid glycoprotein following previously published methods.^20,37^

## RESULTS

Overall, 265 children were enrolled in the study at birth and 240 were available for analysis. Gender was equally represented (male = 49.2%). Median LAZ scores at 7, 15, and 24 months of age were −1.22, −1.74, and −1.98, respectively. Table 1 describes the summary statistics of the participants. Of 240 children, 222 children provided stool samples which were unassociated with diarrhea. Of the 627 stool samples collected (*N* = 222 children), 535, 511, and 577 samples were accompanied by zinc, ferritin, and sTfR values, respectively.

**Table 1:** Descriptive characteristics of the participants at 7, 15, and 24 months

Variables	7 months	15 months	24 months	*P* value
Gender, *n* (%)				
Male	118 (49.2%)	–	–	–
Female	122 (50.8%)	–	–	–
LAZ, median (IQR)	−1.22 (−1.87, −0.58)	−1.74 (−2.42, −1.15)	−1.98 (−2.60, −1.30)	< 0.001
WAMI*, median (IQR)	0.55 (0.48, 0.63)	–	–	< 0.001
CRP (mg/L),† median (IQR)	0.95 (0.40, 2.90)	0.60 (0.30, 2.20)	0.60 (0.15, 1.70)	0.002
AGP (mg/dL),† median (IQR)	89.00 (68.75, 115.25)	86.00 (69.00, 109.00)	72.00 (55.00 −95.00)	< 0.001
Zinc (mmol/L),† median (IQR)	11.00 (9.90, 12.20)	11.00 (10.10, 12.10)	11.80 (10.70, 13.00)	< 0.001
Zinc (mmol/L),† median (IQR)‡	11.30 (10.30, 12.40)	11.23 (10.30, 12.20)	11.80 (10.96, 13.21)	< 0.001
Ferritin (μg/L),† median (IQR)	30.50 (16.00, 48.80)	12.80 (6.83, 21.80)	8.00 (4.80, 14.50)	< 0.001
Ferritin (μg/L),† median (IQR)‡	25.90 (12.82, 42.35)	10.69 (5.67, 18.11)	7.18 (4.70, 13.95)	< 0.001
sTfR (μg/mL),† median (IQR)	5.22 (4.37, 6.75)	7.16 (5.21, 9.55)	6.89 (5.26, 10.77)	< 0.001
Dietary zinc (mg/day),† median (IQR)	–	0.93 (0.59, 1.46)	1.69 (1.15, 2.27)	< 0.001
Dietary iron (mg/day),† median (IQR)	–	1.02 (0.60, 1.63)	2.09 (1.55, 2.95)	< 0.001
Dietary protein (g/day),† median (IQR)	–	8.07 (4.94, 12.00)	14.96 (10.36, 19.07)	< 0.001

AGP = alpha-1-acid glycoprotein; CRP = C-reactive protein; IQR = interquartile range; LAZ = length-for-age *z*-score; sTfR = soluble transferrin receptor.

* The WAMI score (ranging from 0 to 1) is a measure of household socioeconomic status, including access to improved water, sanitation and hygiene; assets; maternal education; and income. Here, W is for water, sanitation and hygiene, A is for assets, M is for maternal education, and I is for income.^38^

† Normal ranges: CRP, ≤ 10 mg/L^20^; AGP, ≤ 100 mg/dL^20^; Zinc, ≥ 9.9 mmol/L^20^; Ferritin, > 12 μg/L^20,39^; sTfR, < 8.3 μg/mL^39^; Dietary Zinc, 3 mg/day^40^; Dietary Iron, 7 mg/day^40^; and Dietary Protein, 13 g/day.^40^

‡ Adjusted for elevated CRP and elevated AGP by mathematical correction.

### Prevalence of stunting, iron deficiency, and zinc deficiency.

**Figure 1.**
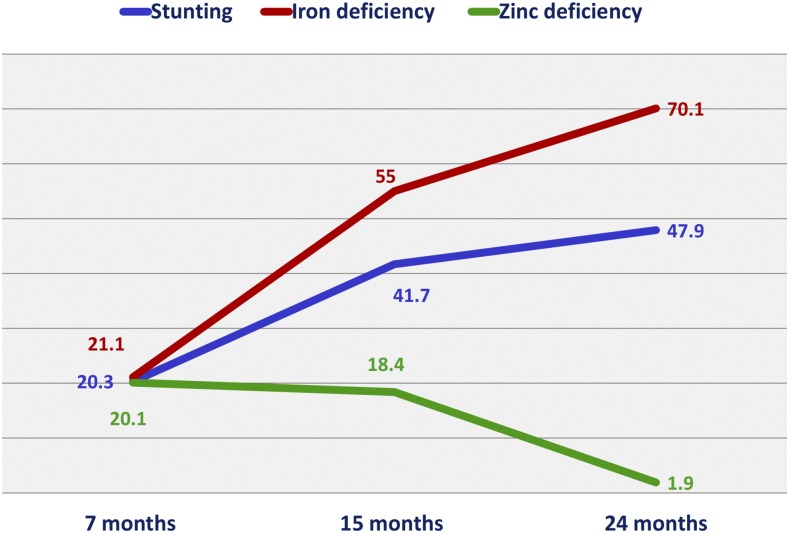
Prevalence of stunting, iron deficiency, and zinc deficiency by months of age. This figure appears in color at www.ajtmh.org.

The prevalence of stunting, iron deficiency, and zinc deficiency by months of age are presented in Figure 1. The prevalence of stunting and iron deficiency increased with age, but zinc deficiency prevalence decreased from 7 to 24 months of age. Prevalence of stunting was 47.9% at 24 months of age, and there was no significant difference between the genders (*P* = 0.144). At 24 months of age, iron deficiency prevalence was 70.1%, whereas the prevalence of zinc deficiency was only 1.9%.

### Fecal marker distribution and categorization.

**Figure 2.**
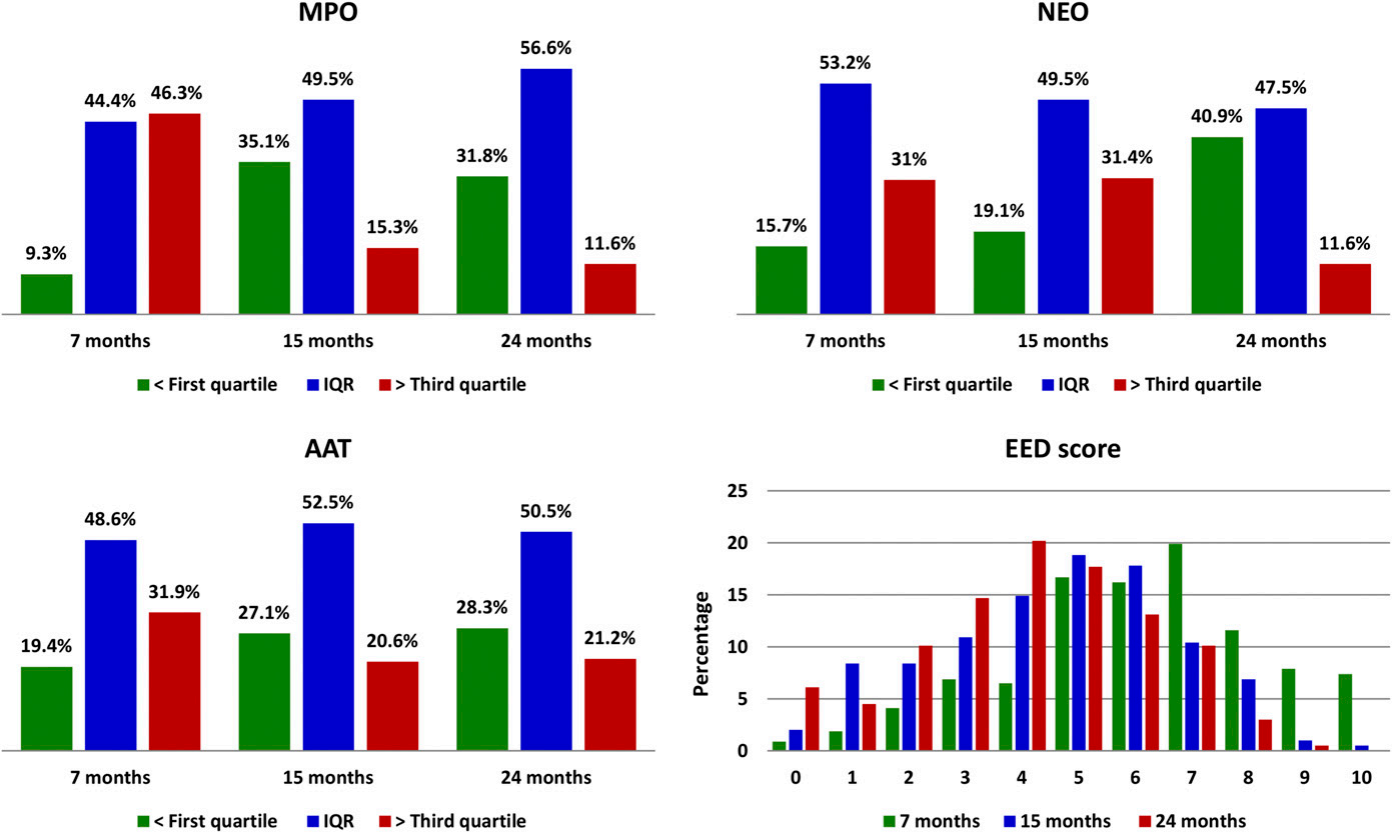
Distribution of fecal markers category and environmental enteric dysfunction (EED) disease activity score by month of age. First three panels of the figure indicate the percentage of samples those have fecal marker values in first quartile, within interquartile range (IQR) ranges, and in third quartile for that individual fecal marker. Panel 4 illustrates the EED score category distribution by months of age, which represents the percentage of samples having EED score ranging from 0 to 10 at 7, 15, and 24 months of age. This figure appears in color at www.ajtmh.org.

The distribution of AAT, MPO, and NEO (*N* = 222) is presented in Table 2. The distribution of fecal markers category and EED disease activity score by month of age are presented in Figure 2. In this cohort of children, approximately 60%, 71%, and 97% of samples were above values considered normal in nontropical settings for AAT (< 0.27 mg/g), MPO (< 2,000 ng/mL), and NEO (< 70 nmol/L), respectively. Median values for AAT, MPO, NEO, and EED score were 0.33 mg/g, 3,895.42 ng/mL, 890.81 nmol/L, and 5, respectively. Table 3 depicts the pairwise examination of Spearman’s correlation between AAT, MPO, and NEO concentrations in the stool. Correlations between the fecal markers were found to be low. The strongest correlation (p = 0.21) were observed between AAT and MPO.

**Table 2:** Distribution of fecal markers and EED disease activity score

	AAT (mg/g)	MPO (ng/mL)	NEO (nmol/L)	EED score (0–10)
*n* (samples)	627	625	627	616
First quartile	0.18	1,563.76	331.57	3
Median	0.33	3,895.42	890.81	5
Third quartile	0.62	8,432.82	2,089.04	7

AAT = alpha-1-anti-trypsin; EED = environmental enteric dysfunction; MPO = myeloperoxidase; NEO = neopterin.

**Table 3:** Pairwise examination of Spearman’s correlation between AAT, MPO, and NEO

	AAT	MPO	NEO
AAT	1		
MPO	0.21*	1	
NEO	0.08*	0.02	1

AAT = alpha-1-anti-trypsin; MPO = myeloperoxidase; NEO = neopterin.

* *P* value < 0.05. The concentrations of these analytes were very weakly correlated.

### Association of fecal markers with zinc and iron status.

Fecal levels of MPO and NEO were not associated with the zinc and iron status of the children in this cohort in univariate analysis. Also, no association was found between fecal levels of AAT and plasma zinc or sTfR levels. However, after adjustment with age and gender, AAT levels were found to be significantly associated with plasma ferritin concentration (Table 4). Children with high AAT levels had significant association with lower ferritin value than those with low AAT at 2 years of age (coefficient = −5.85; 95% confidence interval (CI) = −11.23 to −0.47; *P* value = 0.03).

**Table 4:** Association between fecal markers of EED and plasma ferritin levels during first 2 years of life using generalized estimating equation

Variables	Unadjusted β (95% CI)	*P* value	Adjusted β (95% CI)*	*P* value
Age in days	−0.05 (−0.06, −0.03)	< 0.001	−0.05 (−0.06, −0.03)	< 0.001
Gender	−6.01 (−11.97, −0.05)	0.048	−5.12 (−10.33, 0.09)	0.05
AAT	0.26 (−0.05, 4.33)	0.90	−5.85 (−11.23, −0.47)	0.03†
MPO	0.00‡	0.21	0.00‡	0.05
NEO	0.001 (0.000, 0.002)	0.20	−0.001 (−0.003, 0.000)	0.13
EED score	2.04 (0.45, 3.63)	0.01	1.87 (−0.31, 4.05)	0.09

AAT = alpha-1-anti-trypsin; CI = confidence interval; EED = environmental enteric dysfunction; MPO = myeloperoxidase; NEO = neopterin. Plasma ferritin value was the outcome.

* Variables included in adjusted model were age in days, female gender, AAT, MPO, and NEO.

† *P* value < 0.05.

‡ The coefficient value is very small to report.

## DISCUSSION

According to our study findings, high fecal levels of AAT were associated with decreased ferritin values among children aged less than 2 years living in a slum in Baunibadh area of Mirpur, Bangladesh. Studies have documented that AAT alters iron metabolism in human body.^41^ Alpha-1-anti-trypsin is an acute-phase protein that prevents transferrin from binding to its receptor transferrin receptor.^42^ Synthesis of transferrin receptor and ferritin is regulated bidirectionally in our body.^41,42^ This might be the mechanism of inverse association between AAT and ferritin values. Furthermore, AAT is a marker of protein loss^16^ and ferritin itself is a protein. The negative association between AAT and ferritin values might imply the significance of enteric protein loss in contributing to reduced ferritin values among the children of this cohort living in the poor settings of Mirpur. Although there is no substantial evidence on ferritin loss due to increased gut permeability in human, a previous animal model study conducted on rats strongly suggested that larger macromolecules such as ferritin can move directly, traversing the cells of epithelial lining if intestinal barrier function is lost.^43^ Based on this finding, we hypothesize that our result may be indicative of intestinal ferritin loss due to disruption of gut barrier function resulting from EED during first 2 years of life in this cohort of children. This finding is also inconsistent with a previous study that reported negative relationship between intestinal permeability and serum ferritin concentrations in infants and young children,^44^ indicating AAT as a useful marker of intestinal permeability.

Neither MPO nor NEO was associated with zinc and iron status during the observed period of this analysis. The lack of significant relationship with MPO and NEO might be a reflection of our small sample size. Moreover, plasma zinc concentration is not a reliable biomarker for assessing zinc status; therefore, it possibly hampered our ability to assess the association of zinc status with the fecal markers. Environmental enteric dysfunction disease activity score was formulated by using fecal marker data to demonstrate the intestinal dysfunction more accurately.^12,45^ In the present study, EED score was not associated with zinc or iron status of the children at 7, 15, and 24 months of age. Small sample size can be attributed to this lack of association between EED score and zinc or iron status. Future studies should investigate the relationship prospectively in larger study population.

This study reports that almost half of the children among the participants are stunted at 2 years of age. Such finding delineates the dire burden of chronic malnutrition in the country and highlights the further risk of increasing morbidity and mortality among under-2 children. The prevalence of stunting that we have found in this study is in accordance with the national prevalence. Nationally, 46% of the children aged 18–23 months are stunted in Bangladesh.^4^ Moreover, national data for zinc and iron deficiency are only available for under-5 children in Bangladesh, but not for children younger than 2 years.^20^ According to the latest National Micronutrients Survey, 44.6% and 10.7% under-5 children are zinc and iron deficient, respectively.^20^ In contrast to that, prevalence of iron deficiency was much higher in this cohort of children at 24 months of age. Seven in every 10 under-2 children are iron deficient in Bauniabadh area, depicting serious epidemic of iron deficiency in this area. Surprisingly, the prevalence of zinc deficiency was very low among the children enrolled in this study. Approximately 2% of children were zinc deficient at 2 years of age. Although the prevalence is low in contrast to prior surveys in the same area, our study result regarding plasma zinc concentrations coincides with the findings of National Micronutrient Survey conducted in 2011–12.^20^ Nationally, the mean zinc concentration for preschool children in serum was 10.25 mmol/L. Furthermore, the average zinc concentration for preschool children from urban areas was 11.02 mmol/L, which is similar to our study findings.^20^ But still there is a need to understand more about the low prevalence of zinc deficiency in this cohort of children. However, both the prevalence of stunting and iron deficiency increased with age, wherever zinc deficiency prevalence decreased with increasing age.

The present study includes repeated fecal marker data of the same child at 7, 15, and 24 months of age. Myeloperoxidase and NEO marker levels significantly reduced with increasing age, but AAT levels remain almost same at 15 and 24 months of age (Figure 2). All the fecal markers were highly elevated in the children of this study in comparison to developed countries.^13^ This finding indicates widespread intestinal inflammation and increased intestinal permeability among children aged less than 2 years in the study area. The evidence of highly prevalent iron deficiency and increased intestinal permeability among the children of this cohort supports our finding of inverse relationship between ferritin and AAT. This finding suggests that there might have been an impact of EED on ferritin loss in children during the first 2 years of life.

In conclusion, fecal markers of EED were not associated with zinc status among under-2 children residing in a slum area of Bangladesh. Elevated AAT levels were associated with decreased ferritin values during first 2 years of life. It implies the importance of enteric protein loss in contributing to reduced ferritin values during this critical period of life. This finding may help to develop feasible intervention for preventing ferritin loss due to increased level of AAT. It also implicates the importance of further research regarding contribution of EED on micronutrient status among children.
